# Knowledge and Awareness of Glaucoma Among Medical Students in Aseer Region, Saudi Arabia

**DOI:** 10.7759/cureus.93765

**Published:** 2025-10-03

**Authors:** Abdulrahman Alamri, Hanan A AlKaabi, Ameera T Alzahrani, Atheer H AlMatar, Dalia S Almosleh, Fahad M Wadai, Khadija T Habib, Mohammed E Nizam, Nada M Asiri, Ruba O Alansari, Sabah M Alshahrani, Salem A Alshehri, Yara A Alshehri, Yara A AIziyad

**Affiliations:** 1 College of Medicine, King Khalid University, Abha, SAU; 2 College of Medicine, Imam Abdulrahman Bin Faisal University, Dammam, SAU; 3 Department of Ophthalmology, Aseer Central Hospital, Abha, SAU; 4 College of Medicine, Umm Al-Qura University, Makkah, SAU; 5 College of Medicine, King Saud University, Riyadh, SAU; 6 College of Surgery, King Khalid University, Abha, SAU

**Keywords:** awareness, glaucoma, medical education, medical students, saudi arabia

## Abstract

Background

Glaucoma is one of the causes of irreversible blindness. Early diagnosis and intervention are critical due to the disease's asymptomatic nature in its early stages. As future healthcare professionals, medical students are pivotal in public awareness and education. This study aimed to assess the glaucoma knowledge level among King Khalid University medical students.

Methods

A cross-sectional study was conducted among medical students at King Khalid University between March and May 2025. A sample size of 385 was calculated using Richard Geiger’s formula with an estimated proportion of 50%. Four hundred eighty-one students responded to a self-administered, Arabic questionnaire distributed via Google Forms (Google, Mountain View, CA, US). The tool was adopted from the literature, validated, and piloted for clarity. Data were analyzed using SPSS version 26 (IBM Corp., Armonk, NY, US).

Results

Among the 481 participants, 275 (57%) were female, with a median age of 22 years. Most respondents, 416 (86%), had heard of glaucoma, and 455 (95%) acknowledged the necessity for early treatment. Only 58 (12%) had previously undergone glaucoma screening. Four hundred and four (91.5%) believed that glaucoma can lead to blindness, although 253 (53%) incorrectly believed that glaucoma-induced blindness is reversible. Awareness of risk factors varied: 425 (88%) identified diabetes and hypertension, 452 (94%) recognized age > 60 years, and 252 (52%) acknowledged the hereditary nature of the disease. Symptoms such as headache (448 (93%)) and blurred vision (448 (93%)) were commonly known, but only 200 (42%) understood that glaucoma can be asymptomatic. Only 157 (33%) correctly identified optic nerve damage as the cause. Overall, 217 (54.9%) of students demonstrated good knowledge. Higher knowledge levels were significantly associated with older age (p < 0.001), source of information (p < 0.001), and intention to seek immediate medical attention for eye problems (p = 0.047). No significant association was found between gender and knowledge level (p = 0.6).

Conclusion

Although most students were aware of glaucoma and its major consequences, significant gaps exist in understanding its asymptomatic nature, causes, and treatment modalities. Educational interventions are needed to strengthen glaucoma knowledge among future healthcare professionals in Saudi Arabia.

## Introduction

Glaucoma encompasses a group of progressive optic neuropathies characterized by irreversible damage to the optic nerve, frequently-but not exclusively-associated with elevated intraocular pressure (IOP). It is recognized globally as the second leading cause of irreversible blindness [1]. Recent epidemiological estimates indicate that more than 76 million individuals were affected by glaucoma worldwide in 2020, with projections suggesting an increase to over 111 million by the year 2040 [2].

In the Kingdom of Saudi Arabia, a cross-sectional study conducted in 2019 in the Riyadh governorate reported a glaucoma prevalence of 5.6% [3]. This prevalence is comparable to findings from neighboring countries such as Qatar and Iran [4,5].

Multiple risk factors have been implicated in the development of glaucoma. These include elevated IOP, age above 40 years, diabetes mellitus, systolic hypertension, and a positive family history of glaucoma [6].

A major challenge in the management of glaucoma is its typically asymptomatic course in the early stages, which has led to its description as "the silent thief of sight" [7]. Given the irreversible nature of vision loss due to glaucoma, early detection and timely intervention are essential, highlighting the critical need for disease awareness among future healthcare providers [8].

As future physicians and public health educators, medical students are pivotal in the early detection and awareness campaigns related to glaucoma. Studies assessing their level of awareness have shown varying degrees of knowledge. Most students know glaucoma and understand it is a treatable condition [9,10]. However, the depth of their knowledge remains inconsistent. For example, a study from Punjab reported that 45% of medical students correctly identified glaucoma as the leading cause of irreversible blindness, and 48% recognized its genetic predisposition [11]. Similarly, in Southern Nigeria, medical students displayed good general knowledge of the disease, but confidence in performing ocular examinations was low [9].

A study conducted in Ghana revealed that while the term "glaucoma" was widely recognized among healthcare students, substantial gaps existed in understanding key elements such as risk factors, disease pathophysiology, and available treatment options [12]. Notably, many students lack adequate awareness regarding the asymptomatic nature and irreversibility of primary open-angle glaucoma (POAG). Only 30% of students in the Punjab study were aware that POAG is typically asymptomatic, and less than half recognized the irreversible nature of glaucoma-related vision loss [11]. Another study reported that only 12.2% of students correctly identified POAG as asymptomatic [13]. These findings suggest that enrollment in a health sciences program does not inherently ensure a comprehensive understanding of glaucoma [10].

Despite the clinical importance of glaucoma, data regarding knowledge levels among medical students in Saudi Arabia remain limited, particularly in the southern regions such as Aseer. Therefore, this study aims to assess the knowledge of glaucoma among medical students at King Khalid University.

## Materials and methods

Study design, setting, and population

A cross-sectional study was conducted among the medical students in King Khalid University of Saudi Arabia between March and May 2025. We excluded students with cognitive problems who could not respond to the questionnaire and those who refused to participate in the study. Non-students, physicians, and men and women from colleges other than Medicine, Pharmacy, Dentistry, and Nursing were also excluded.

Sample size calculation

The sample size was calculated based on the Richard Geiger formula of sample size estimation [14]. With a margin of error of 5%, a confidence level of 95%, and an estimated proportion of attributes of 50%, 385 medical students were selected.

Sampling technique and study instrument

Data were collected conveniently through Google Forms (Google, Mountain View, CA, US) distributed through social media platforms using an Arabic self-administered questionnaire. The questionnaire was developed based on literature from an open-access study [15], and we used it following its open-access license. It underwent face validity evaluation by family medicine specialists.

A pilot study involving 20 students was conducted to assess the clarity and comprehensibility of the questionnaire. Feedback from the pilot study led to minor refinements, and responses from the pilot study were excluded from the final analysis.

The finalized version consisted of two sections; the first included demographic characteristics such as age, gender, sources of information about glaucoma, duration till consulting a physician in case of having eye problems, and history of screening for glaucoma before. The second assessed the knowledge of glaucoma regarding its signs and symptoms, risk factors, and consequences.

Statistical analysis

Data were gathered in Microsoft Excel (Microsoft Corp., Redmond, WA, US), cleaned, and analyzed using SPSS version 26 (IBM Corp., Armonk, NY, US). The normality of continuous variables was assessed using histograms and the Kolmogorov-Smirnov test. Descriptive statistics included frequencies and percentages for categorical variables and median with interquartile range (IQR) for continuous variables. Correct answers were coded as one for the knowledge score, incorrect answers were coded as zero, and the total score was calculated. Participants who scored ≥ the mean were considered to have good knowledge of glaucoma. Pearson's Chi-squared and Wilcoxon rank sum tests were performed to identify predictors of good knowledge of glaucoma. Statistical significance was set at p < 0.05.

Ethical considerations

The study was approved by the scientific research ethics committee of King Khalid University (Institutional Review Board (IRB) registration number 2025-708). Informed consent was obtained electronically via a mandatory consent checkbox in Google Forms before participants could proceed with the survey. Data confidentiality was maintained by anonymizing responses, with no personally identifiable information collected.

## Results

A total of 481 participants were included in the study. The majority were women, 275 (57%), while men accounted for 206 (43%). The median age was 22.00 years (IQR: 20.00, 23.00). Regarding the source of information about glaucoma, 245 (51%) participants reported doctors as their primary source, followed by 123 (26%) who relied on social media, 86 (18%) who referred to books and magazines, and 27 (5.6%) who mentioned friends.

Regarding health-seeking behavior, 383 (80%) participants reported that they would immediately consult a physician in case of eye problems. Additionally, 72 (15%) reported consulting a physician within a day or two, and 26 (5.4%) would do so after more than two days. Despite this awareness, only 58 (12%) participants reported having been screened for glaucoma before (Table 1).

**Table 1:** Demographic characteristics of study participants IQR: interquartile range

Characteristic	n (%); median (IQR)
Gender	
Female	275 (57%)
Male	206 (43%)
Age (years)	22.00 (20.0, 23.0)
Source of information about glaucoma	
Books & magazine	86 (18%)
Doctor	245 (51%)
Friends	27 (5.6%)
Social media	123 (26%)
Duration till consulting a physician in case of having eye problems	
Immediately	383 (80%)
A day or two	72 (15%)
After 2 days	26 (5.4%)
History of screening for glaucoma before	
No	423 (88%)
Yes	58 (12%)

Most participants, 414 (86%), had previously heard of glaucoma, and a significant proportion, 457 (95%), acknowledged the necessity of early treatment. In addition, 443 (92%) recognized the importance of routine eye examinations starting at 40 for early detection, and 440 (91%) correctly identified that glaucoma can lead to blindness. However, only 255 (53%) were aware that blindness resulting from glaucoma is irreversible.

Concerning symptoms, 399 (83%) correctly reported eye pain as a symptom, 279 (58%) recognized nausea and vomiting, and 448 (93%) identified headache and blurred vision as associated features. Notably, 202 (42%) correctly understood that glaucoma can be asymptomatic.

More than half of the participants, 250 (52%), were aware of the hereditary nature of the disease, and 423 (88%) identified chronic conditions such as diabetes mellitus and hypertension as risk factors. Approximately 452 (94%) acknowledged that individuals above 60 are at higher risk, and 366 (76%) recognized previous eye trauma or surgery as contributing factors. Additionally, 294 (61%) correctly identified refractive errors (such as farsightedness and high myopia) as risk factors. Furthermore, 443 (92%) agreed that early screening prevents complications.

Despite this, 260 (54%) correctly believed that surgery is the only treatment for glaucoma. While a high proportion, 443 (92%), understood the importance of informing healthcare providers about a glaucoma diagnosis to avoid medications that may worsen the condition, only 158 (33%) correctly identified optic nerve damage as the underlying cause of glaucoma, as shown in Table 2.

**Table 2:** Knowledge of glaucoma among study participants Data are presented as n (%) *Correct answer

Characteristic	No	Yes
Heard about glaucoma before	65 (14%)	416 (86%)*
Glaucoma should be treated early	26 (5.4%)	455 (95%)*
Regular check-ups of the eyes from the age of 40 are necessary to detect glaucoma early	40 (8.3%)	441 (92%)*
Glaucoma will lead to blindness	41 (8.5%)	440 (91%)*
The blindness resulting from glaucoma can be treated	228 (47%)	253 (53%)*
Red eye is a symptom of glaucoma	194 (40%)	287 (60%)*
Symptoms of glaucoma include eye pain	84 (17%)	397 (83%)*
Nausea and vomiting are one of the symptoms of glaucoma	201 (42%)	280 (58%)*
Headache and blurred vision are one of the symptoms of glaucoma	33 (6.9%)	448 (93%)*
Glaucoma is an asymptomatic disease	281 (58%)	200 (42%)*
Glaucoma is a hereditary disease	229 (48%)	252 (52%)*
Chronic diseases (diabetes mellitus, hypertension) are risk factors for glaucoma	56 (12%)	425 (88%)*
People more than 60 years of age are more susceptible to glaucoma	29 (6.0%)	452 (94%)*
Surgeries or a previous eye injury are risk factors for glaucoma	114 (24%)	367 (76%)*
Farsightedness and severe nearsightedness are risk factors for glaucoma	188 (39%)	293 (61%)*
Early screening helps avoid complications of glaucoma	37 (7.7%)	444 (92%)*
The only treatment for glaucoma is surgical	261 (54%)	220 (46%)*
It is important to tell your doctor you have glaucoma to avoid medication that can exacerbate the glaucoma	39 (8.1%)	442 (92%)*
Glaucoma results from		
Damage to the optic nerve*	157 (33%)	
Blurry lens	218 (45%)	
I do not know	106 (22%)	

**Figure 1:**
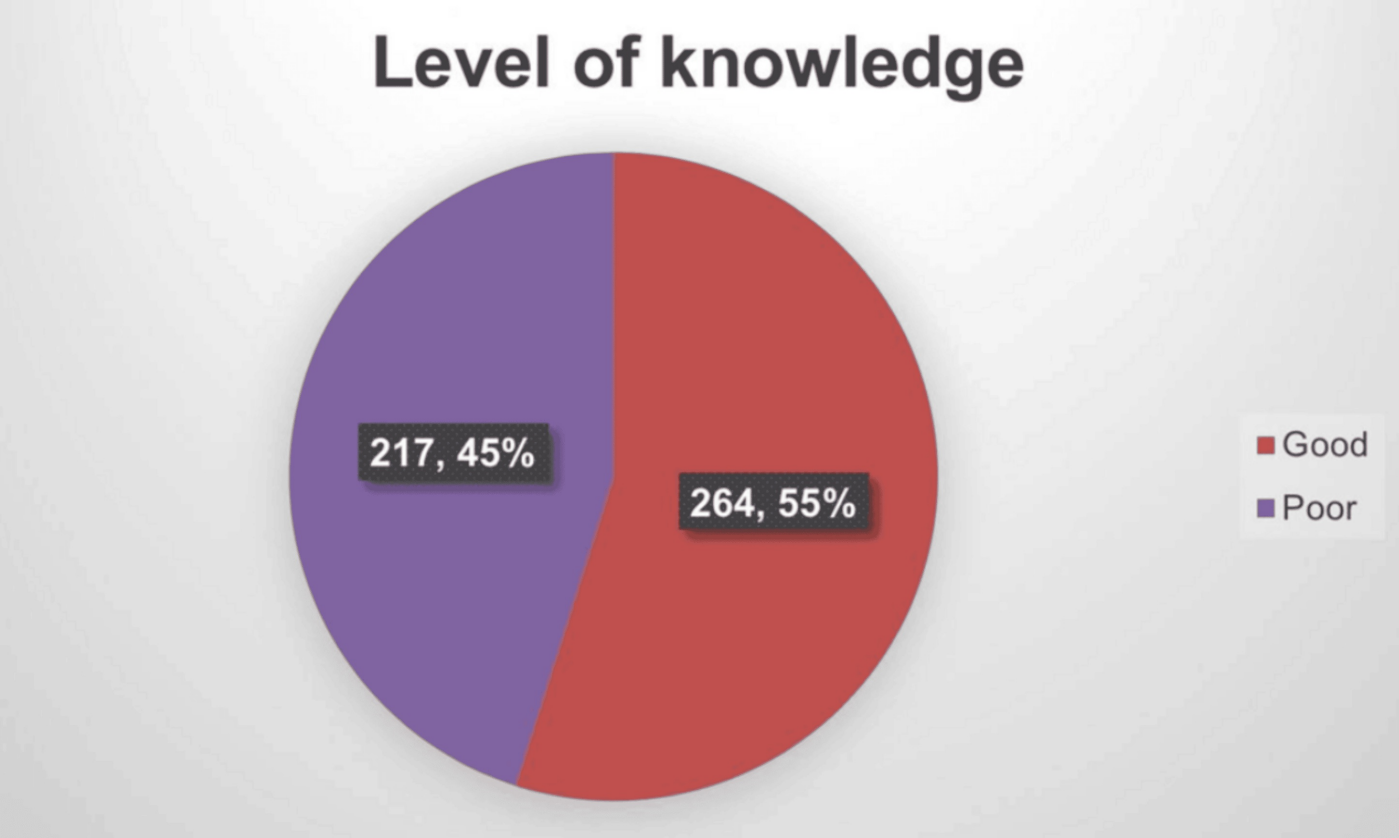
Level of knowledge regarding glaucoma

The mean knowledge score was 13.9 ± 2.5, with a median of 14, minimum of 6, and maximum of 19. However, 264 (54.9%) had good knowledge of glaucoma, while 217 (45.1%) had poor knowledge (Figure 1).

Gender was not significantly associated with the level of knowledge regarding glaucoma (p = 0.6); however, women had higher knowledge than men (56% vs. 53.4%). However, participants with a higher level of knowledge had a significantly greater median age than those with lower knowledge levels (p < 0.001). Participants who reported obtaining information about glaucoma from books and magazines (62.8%) had good knowledge compared to 62% of those who got their information from doctors (p < 0.001), 33.3% from friends, and 39.8% from social media.

Additionally, 57.7% of those who stated they would consult a physician immediately upon experiencing eye problems had significantly greater knowledge than 43.1% of those who consult within a day or two and 46.2% of those who consult after two days, respectively (p = 0.047). Similarly, participants who had screened for glaucoma before had higher levels of good knowledge than those who did not (62.1% vs. 53.9%); however, the association was not statistically significant (p = 0.2) (Table 3).

**Table 3:** Association between demographic characteristics and the level of knowledge regarding glaucoma ^1^n (%); median (IQR) ^2^Pearson's Chi-squared test; Wilcoxon rank sum test IQR: interquartile range

Characteristic	Good, N = 264^1^	Poor, N = 217^1^	Test statistic	p-value^2^
Gender			0.7	0.6
Female	154 (56%)	121 (44%)		
Male	110 (53%)	96 (47%)		
Age (years)	22.0 (21.0, 23.0)	21.0 (20.0, 23.0)	230,880	<0.001
Source of information about glaucoma			27.5	<0.001
Books & magazine	54 (63%)	32 (37%)		
Doctor	152 (62%)	93 (38%)		
Friends	9 (33%)	18 (67%)		
Social media	49 (40%)	74 (60%)		
Duration till consulting a physician in case of having eye problems			8.9	0.047
Immediately	221 (58%)	162 (42%)		
A day or two	31 (43%)	41 (57%)		
After 2 days	12 (46%)	14 (54%)		
History of screening for glaucoma			0.2	0.2
No	228 (54%)	195 (46%)		
Yes	36 (62%)	22 (38%)		

Among the examined factors, only the source of information about glaucoma showed a significant association with the knowledge level. Students who reported friends (odds ratio (OR): 3.1, 95% confidence interval (CI): 1.4, 7.7, p = 0.009) or social media (OR: 2.4, 95% CI: 1.5, 3.9, p < 0.001) as their primary sources demonstrated higher knowledge scores compared to those who cited doctors as their main source of information (Table 4).

**Table 4:** Determinants of the level of knowledge regarding glaucoma OR: odds ratio; CI: confidence interval

Characteristic	OR	95% CI	p-value
Gender			
Female	—	—	
Male	1.4	0.9, 2.1	0.08
Age	1	0.9, 1.1	0.7
Source of information about glaucoma			
Doctor	—	—	
Books & magazine	0.9	0.6, 1.6	0.9
Friends	3.1	1.4, 7.7	0.009
Social media	2.4	1.5, 3.9	<0.001
Duration till consulting a physician in case of having eye problems			
Immediately	—	—	
A day or two	1.4	0.8, 2.4	0.2
After 2 days	1.4	0.6, 3.2	0.4
History of screening for glaucoma			
No	—	—	
Yes	0.7	0.4, 1.3	0.3

## Discussion

Glaucoma is a group of eye disorders characterized by progressive optic nerve damage, often associated with elevated IOP, and is recognized as the second leading cause of irreversible blindness worldwide [1]. It is estimated that over 76 million people were affected by glaucoma globally in 2020, and the number is expected to rise to over 111 million by 2040 [2]. One of the most challenging aspects of glaucoma is its asymptomatic nature in the early stages, earning it the label "the silent thief of sight" [7]. Since early diagnosis and timely management are crucial to preventing permanent vision loss, awareness and understanding of the disease among future healthcare professionals are essential [8]. Medical students, in particular, play a key role in promoting early detection and educating the public. Despite this, limited data exist regarding their level of knowledge and awareness of glaucoma, especially in the Aseer Region of Saudi Arabia. This study evaluated the awareness and understanding of glaucoma among medical students at King Khalid University.

When compared with previous literature, our findings demonstrate relatively higher general awareness among medical students. For instance, a study conducted among medical students in North India found that while 100% knew glaucoma is an eye disease, many were unaware of its silent progression, risk factors, and complications. Only 45% recognized that glaucoma can be asymptomatic, and misconceptions about treatment were common [16]. In our study, 42% correctly identified glaucoma as potentially asymptomatic, which is slightly lower, reflecting a similar trend in insufficient understanding of the disease’s silent nature.

In a Saudi study conducted in Taif City, 58.5% of participants had heard of glaucoma, and 74.5% knew it could lead to blindness [17]. Our study demonstrated higher awareness levels in both categories (86% and 91%, respectively), possibly due to the medical background of our participants. However, both studies shared similarities in that participants often lacked knowledge about glaucoma’s irreversibility and underlying pathology. Moreover, our study revealed that 54% of participants incorrectly believed surgery is the sole treatment for glaucoma, highlighting a significant misconception. This aligns with findings from a study conducted in Jazan, Saudi Arabia, where only 27.8% of participants were aware of treatment options for glaucoma, indicating a substantial gap in public knowledge regarding glaucoma management [18]. Furthermore, a recent study conducted in Abha, Southern Saudi Arabia, reported that while 77.1% of participants had heard of glaucoma, detailed knowledge remained limited. Additionally, the study showed that awareness was significantly higher among participants who consulted a physician immediately upon experiencing eye problems (53.2%) compared to those who delayed consultation (38.7%), and among those who had undergone glaucoma screening (57.1%) versus those who had not (45.8%) (p = 0.042). These findings are consistent with our results, where older students and those with prior screening experiences demonstrated significantly better knowledge levels (p < 0.001 and p = 0.2, respectively) [15]. Another study from Ghana also indicated that health students had high awareness levels but still showed significant knowledge gaps in disease mechanisms and management options [12].

Our results also show an interesting trend in information sources. Students who obtained information from books and magazines demonstrated significantly higher knowledge than those who relied on doctors or social media (p < 0.001). This might indicate that more structured or academically rigorous sources promote better retention and understanding, consistent with the findings from Nigeria, where formal sources like lectures, seminars, and ophthalmologists dominated glaucoma awareness [19]. While 80% of our participants stated they would consult a physician immediately for eye problems, only 12% had ever undergone glaucoma screening. This highlights a disconnect between perceived importance and personal health-seeking behavior, echoing findings from previous studies in Saudi Arabia and other regions [20].

Our results showed an increased level of glaucoma knowledge associated with disease awareness (p < 0.001). Moreover, we found a significantly higher level of knowledge among women (p = 0.042); this is different from the results reported by a previous investigation among the adult population, showing significantly higher awareness in men than women [21].

Despite these valuable insights, this study has several limitations. First, it employed a cross-sectional design, which does not establish causality. Second, the self-reported nature of the questionnaire could introduce bias, particularly social desirability bias. Third, the findings may not be generalizable beyond the Aseer Region or to non-medical populations. Furthermore, we acknowledge that formal psychometric validation (e.g., reliability and construct validity) was not performed. Lastly, although the sample size was adequate, a more balanced representation from different academic years could have added more depth to our analysis.

## Conclusions

In conclusion, medical students in the Aseer Region of Saudi Arabia demonstrate commendable awareness of glaucoma and its risk factors, but there are evident gaps in detailed clinical knowledge, particularly regarding irreversible outcomes and disease mechanisms. These findings highlight the need to integrate more structured ophthalmologic education into medical curricula and to promote routine screening practices among future healthcare providers.

## Appendices

Supplemental material 1

**Table 5:** Personal data, source of information, and practice of the sampled population

Gender	Male
	Female
Age in years	
Source of information for glaucoma	Doctor
	Friends
	Internet and social media
Duration till consulting physician in case of having eye problems	Immediately
	A day or two
	After 2 days
Did you do screening for glaucoma before	Yes
	No

Supplemental material 2 

**Table 6:** Awareness of the sampled population regarding glaucoma in Aseer region, Saudi Arabia

Did you ever hear about glaucoma?	Yes	No
Do you believe that glaucoma should be treated early?	Yes	No
Do you think regular check-ups of eyes from age 40 are necessary to detect glaucoma early?	Yes	No
Do you think glaucoma will lead to blindness?	Yes	No
Do you think the blindness resulting from glaucoma can be treated?	Yes	No
Do you think red eye is a symptom of glaucoma?	Yes	No
Do you think that symptoms of glaucoma include eye pain?	Yes	No
Do you think nausea and vomiting are one of the symptoms of glaucoma?	Yes	No
Do you think headache and blurred vision are one of the symptoms of glaucoma?	Yes	No
Do you think glaucoma is an asymptomatic disease?	Yes	No
Do you think glaucoma is a hereditary disease?	Yes	No
Do you think chronic diseases like diabetes mellitus and hypertension are a risk factor for glaucoma?	Yes	No
Do you think people more than 60 years of age are more susceptible to glaucoma?	Yes	No
Do you think that surgeries or a previous eye injury is a risk factor for glaucoma?	Yes	No
Do you think farsightedness and severe nearsightedness are risk factors for glaucoma?	Yes	No
Does early screening help avoid complications of glaucoma?	Yes	No
Do you think the only treatment for glaucoma is surgical?	Yes	No
Do you think it is important to tell your doctor you have glaucoma to avoid medication that can exacerbate the glaucoma?	Yes	No
Glaucoma results from?		
Blurry lens		
Damage to optic nerve		
Do not know		
Total score (mean ± SD)		
